# Spinal Subdural Hematoma Initially Misdiagnosed, Revealed by CT Brain Window Settings

**DOI:** 10.7759/cureus.100443

**Published:** 2025-12-30

**Authors:** Chisato Nakajima, Masahiro Kashiura, Shiho Togo, Hideto Yasuda, Takashi Moriya

**Affiliations:** 1 Department of Emergency and Critical Care Medicine, Saitama Medical Center, Jichi Medical University, Saitama, JPN

**Keywords:** ct imaging, magnetic resonance imaging, spinal subdural hematoma, stroke mimic, window settings

## Abstract

This case demonstrates that meticulous physical examination and optimized computed tomography (CT) windowing are crucial for diagnosing spinal subdural hematoma (SSH) when it mimics stroke. We present a 43-year-old man with a stroke-like onset of unilateral leg weakness that progressed to paraplegia. While a sensory level at T4 strongly suggested a spinal cord lesion, a standard trunk CT was initially interpreted as normal. The diagnosis of a C7-T4 SSH was ultimately established by re-reviewing the CT using brain window settings and was confirmed by magnetic resonance imaging. Despite emergent surgical decompression, the patient had minimal neurological recovery. This report underscores two key lessons: the diagnostic priority of spinal-specific signs (e.g., a sensory level) over misleading motor deficits, and the utility of applying brain window settings as a simple, effective technique to avoid misdiagnosing this critical condition.

## Introduction

Spinal subdural hematoma (SSH) is a rare condition that can cause significant neurological injury and morbidity. It is most commonly associated with anticoagulation therapy, iatrogenic procedures such as lumbar puncture, or trauma [1]. Cases without any identifiable cause, known as idiopathic SSH, are even more uncommon [2]. Patients typically present with an acute onset of severe back pain, followed by progressive motor and sensory deficits [1,3]. Magnetic resonance imaging (MRI) is the gold standard for diagnosing SSH, offering excellent visualization of the hematoma and its effect on the spinal cord [1]. While multidetector computed tomography (CT) is the first-line modality for evaluating spinal trauma, its role in detecting non-traumatic spinal hemorrhage is considered complementary [4].

However, the diagnosis of SSH can be challenging, particularly when the clinical presentation is atypical. SSH can mimic more common neurological emergencies such as stroke, especially when presenting with unilateral weakness, as documented in previous reports [2,3,5-7]. This can potentially lead to diagnostic delays. Furthermore, on CT, an acute subdural hematoma can appear isodense to the spinal cord or otherwise be subtle. While the utility of CT with soft tissue window settings for detecting perispinal hematoma has been reported, the optimal approach for identifying subdural hematomas remains unclear, presenting a significant diagnostic pitfall [8]. For acute hematomas, CT attenuation values are generally reported to be approximately 55-65 HU. Therefore, using a brain window, such as WL 35 and WW 80, may offer higher detection sensitivity than a soft tissue window [9,10].

Here, we present a case of idiopathic spinal subdural hematoma in a patient who initially presented with acute unilateral leg weakness, mimicking stroke. In this case, the hematoma was identified by applying brain window settings to a plain CT. The purpose of this report is to emphasize the value of a systematic physical examination in the differential diagnosis of acute paralysis and to highlight the diagnostic utility of applying brain window settings to CT scans for detecting these elusive hematomas.

## Case presentation

A 43-year-old man, with a past medical history significant only for right testicular tumor resection in childhood and no regular medications or known allergies, developed sudden-onset left leg weakness and chest discomfort. He was transported by ambulance to a local hospital. At the initial hospital, while the patient exhibited paralysis and sensory deficits in the left lower extremity, other signs characteristic of stroke, including upper limb paralysis, facial palsy, and dysarthria, were notably absent. A plain CT scan of the head and trunk performed one hour after onset showed no acute abnormalities. Given the unexplained paralysis, he was subsequently transferred to our facility for further specialist evaluation. The patient’s leg paralysis progressed bilaterally during ambulance transport.

Upon arrival at our department (three hours after onset), his vital signs were stable. The neurological examination was remarkable for a clear sensory level to touch and temperature at the T4 dermatome, with diminished sensation at T5 and absent sensation below T6. Motor examination demonstrated complete paralysis (Medical Research Council grade 0/5) of the lower extremities, while upper extremity strength was normal, and the Barré sign was negative. Urinary retention was also noted. Clinical laboratory tests, including coagulation studies and complete blood count, were all within normal ranges: platelets 22.4 × 10⁴/μL, prothrombin time/international normalized ratio (PT-INR) 0.98, and activated partial thromboplastin time (APTT) 22.8 seconds.

**Figure 1:**
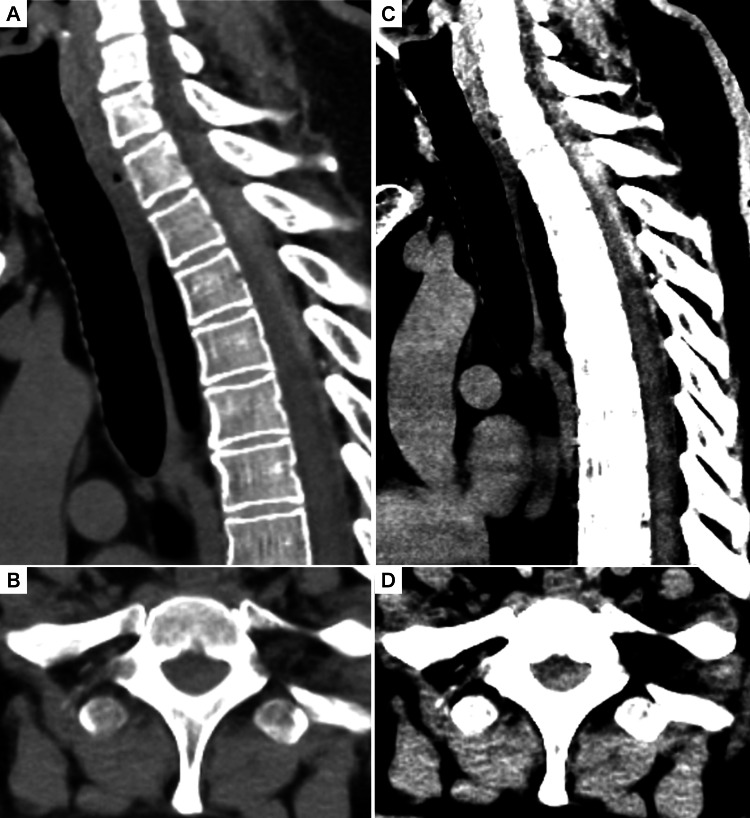
Plain trunk CT (reconstructed from 1-mm source data) (A, B) Sagittal (A) and axial (B) reconstructions of a non-contrast trunk CT on a standard mediastinal window (WL 40, WW 320). The subdural hematoma is poorly defined and nearly isodense with the spinal cord. (C, D) The same sagittal (C) and axial (D) views reviewed with high-contrast brain window settings (WL 35, WW 80).

**Figure 2:**
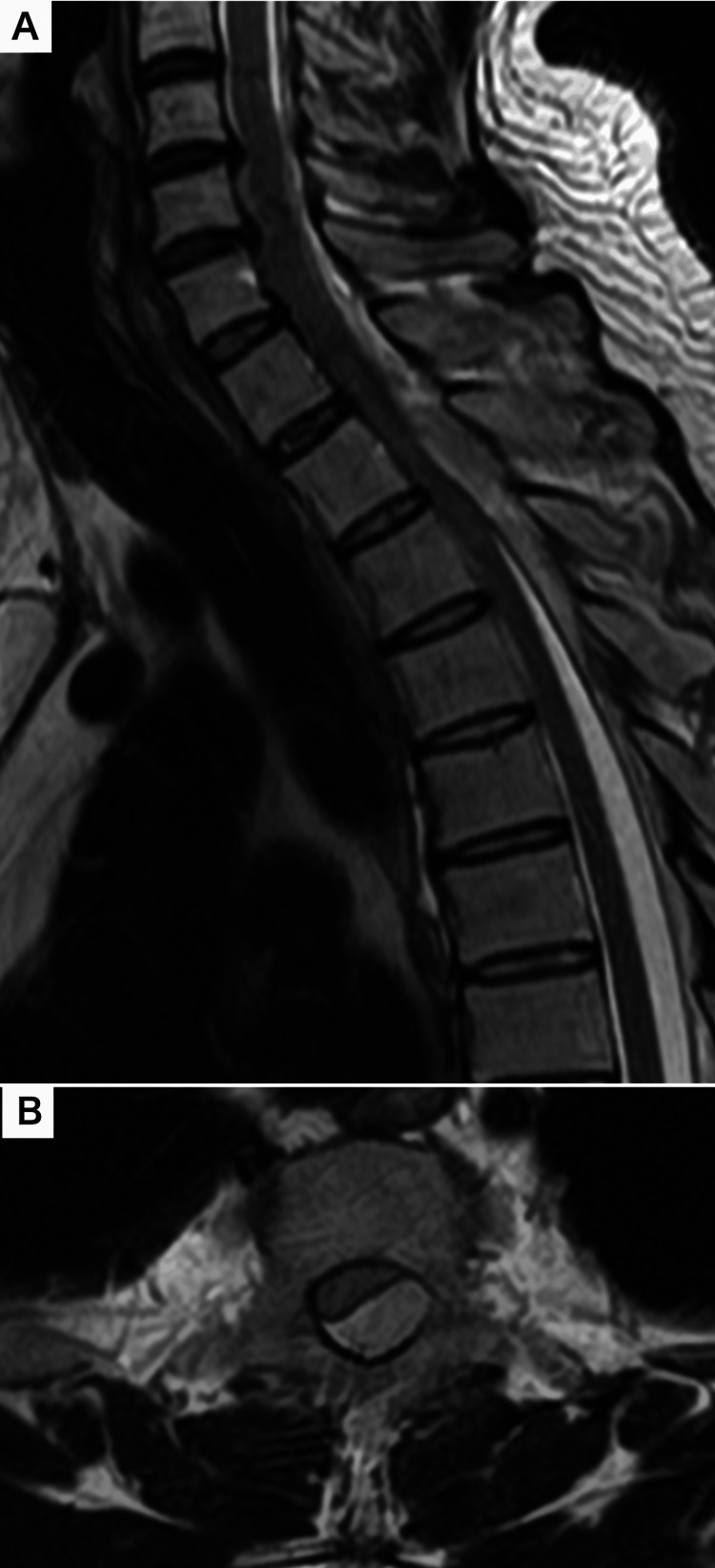
Thoracic spine MRI (T2-weighted image) Sagittal (A) and axial (B) T2-weighted MRI of the spine confirms the acute subdural hematoma, which appears as a hyperintense collection causing significant cord compression.

Review of the imaging from the referring hospital yielded the critical diagnostic clue. While the plain trunk CT had initially been considered unremarkable, re-examination of the images at our facility using brain window settings unveiled a crescent-shaped, high-density collection in the posterior subdural space. This collection extended from C7 to T4, causing significant spinal cord compression (Figure 1). An MRI performed at our hospital four hours after onset confirmed the presence of an acute subdural hematoma at the C7-T4 level, which showed high signal intensity on T2-weighted images (Figure 2).

For management of the acute spinal cord compression, the patient was transferred emergently to a specialized spine center (eight hours after onset). He underwent a T1-T2 laminectomy with partial hemilaminectomies of the left C7 and T3 for hematoma evacuation. Intraoperatively, a dark-red, clot-like hematoma was encountered in the subdural space and successfully evacuated. The postoperative course, however, was marked by minimal neurological recovery; lower extremity paralysis persisted with motor strength of 0/5, and sensory, bladder, and bowel dysfunction remained. On postoperative day 24, he was transferred to a rehabilitation facility for intensive therapy.

## Discussion

This case highlights two critical clinical lessons: first, the importance of a meticulous physical examination in differentiating spinal cord compression from more common causes of acute paralysis, such as stroke, and second, the diagnostic utility of optimizing CT window settings for the detection of acute spinal subdural hematomas. The patient’s initial presentation of unilateral weakness was highly suggestive of a cerebrovascular event, and the definitive radiological finding was only revealed after adjusting the CT review parameters from the standard mediastinal window to a brain window. These aspects deserve further discussion, as they have significant implications for the timely and accurate diagnosis of this rare but critical condition.

The first lesson from this case reinforces the primacy of a systematic physical examination in evaluating acute paralysis. Our patient’s unilateral leg weakness initially pointed toward a cerebrovascular accident, a common diagnostic pitfall for SSH as documented in previous reports [2,3,5-7]. However, a meticulous neurological assessment can uncover findings inconsistent with a cerebral origin. While our patient’s chest discomfort was an early clue, the definitive sign was the clear sensory level at the T4 dermatome. This finding, combined with the early onset of urinary retention, suggests a localized lesion within the spinal cord. Therefore, even when faced with stroke-like motor deficits, the presence of spinal red flags, such as localized pain, a distinct sensory level, or early sphincter disturbance, must prompt urgent consideration and imaging of the spinal cord.

The second lesson from this case demonstrates the diagnostic utility of applying brain window settings to spinal CT scans for spontaneous spinal epidural hematomas, which also present with severe pain and paralysis. According to one report, a mass could be detected in seven of nine cases using CT with standard soft tissue window settings [8]. However, our case of a subdural hematoma illustrates a potential limitation of this approach. An acute subdural hematoma can be thinner and more isodense with the spinal cord, making it difficult to discern even on mediastinal windows. In our patient, the high-density hematoma was clearly delineated only after reviewing the images with high-contrast brain window settings (WL 35, WW 80). This aligns with the principle advocated by Turner and Holdsworth for head CTs, where narrow stroke windows enhance the detection of subtle pathologies missed on default settings [9]. While MRI is the definitive diagnostic modality, its availability can be limited, potentially delaying diagnosis as neurological symptoms progress [1]. Therefore, the ability to identify a spinal hematoma on an immediately accessible CT scan is of great clinical value in the emergency setting. Our case builds upon previous findings by suggesting that when a spinal stroke mimic is encountered and a hematoma is not evident on standard soft tissue CT windows, a simple re-examination with brain window settings should be performed before ruling out hemorrhage.

## Conclusions

In conclusion, this case of idiopathic spinal subdural hematoma underscores two simple yet critical practices for the frontline clinician. First, it highlights the importance of maintaining a high index of suspicion for spinal pathology in any patient with acute paralysis. A systematic search for spinal “red flags,” such as a clear sensory level or localized pain, is essential to avoid the pitfall of prematurely diagnosing a stroke, even when motor deficits are unilateral. Second, it demonstrates that simple adjustments in CT image interpretation, specifically the application of brain or subdural window settings, can serve as a pivotal, no-cost tool for revealing occult spinal hemorrhages.

## References

[1] Moriarty HK, O Cearbhaill R, Moriarty PD, Stanley E, Lawler LP, Kavanagh EC (2019). MR imaging of spinal haematoma: a pictorial review. Br J Radiol.

[2] Wang Y, Zheng H, Ji Y, Lu Q, Li X, Jiang X (2018). Idiopathic spinal subdural hematoma: case report and review of the literature. World Neurosurg.

[3] Kobayashi K, Imagama S, Ando K, Nishida Y, Ishiguro N (2017). Acute non-traumatic idiopathic spinal subdural hematoma: radiographic findings and surgical results with a literature review. Eur Spine J.

[4] Shabani S, Meyer BP, Budde MD, Wang MC (2021). Diagnostic imaging in spinal cord injury. Neurosurg Clin N Am.

[5] Oh SH, Han IB, Koo YH, Kim OJ (2009). Acute spinal subdural hematoma presenting with spontaneously resolving hemiplegia. J Korean Neurosurg Soc.

[6] Matsumoto H, Miki T, Miyaji Y (2012). Spontaneous spinal epidural hematoma with hemiparesis mimicking acute cerebral infarction: two case reports. J Spinal Cord Med.

[7] Patel R, Kumar A, Nishizawa K, Kumar N (2018). Hemiparesis in spontaneous spinal epidural haematoma: a potential stroke imitator. BMJ Case Rep.

[8] Eto F, Tatsumura M, Iwabuchi S, Ogawa T, Mammoto T, Hirano A (2019). Clinical features of spontaneous spinal epidural hematoma. J Rural Med.

[9] Turner PJ, Holdsworth G (2011). Commentary. CT stroke window settings: an unfortunate misleading misnomer?. Br J Radiol.

[10] Chen Y, Cao D, Guo ZQ (2022). The attenuation value within the non-hypodense region on non-contrast computed tomography of spontaneous cerebral hemorrhage: a long-neglected predictor of hematoma expansion. Front Neurol.

